# A Retention-Matching Strategy for Method Transfer in Supercritical Fluid Chromatography: Introducing the Isomolar Plot Approach

**DOI:** 10.1021/acs.analchem.0c05142

**Published:** 2021-04-12

**Authors:** Martin Enmark, Jörgen Samuelsson, Torgny Fornstedt

**Affiliations:** Department of Engineering and Chemical Sciences, Karlstad University, Karlstad SE-651 88, Sweden

## Abstract

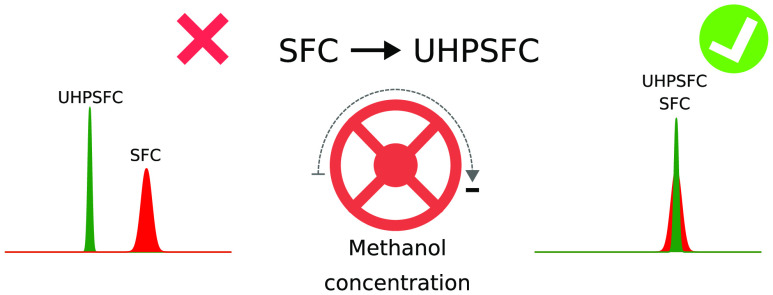

A strategy to match any retention shifts due to increased or decreased pressure drop during supercritical fluid chromatography (SFC) method transfer is presented. The strategy relies on adjusting the co-solvent molarity without the need to adjust the back-pressure regulator. Exact matching can be obtained with minimal changes in separation selectivity. To accomplish this, we introduce the isomolar plot approach, which shows the variation in molar co-solvent concentration depending on the mass fraction of co-solvent, pressure, and temperature, here exemplified by CO_2_–methanol. This plot allowed us to unify the effects of the co-solvent mass fraction and density on retention in SFC. The approach, which was verified on 12 known empirical retention models for each enantiomer of six basic pharmaceuticals, allowed us to numerically calculate the apparent retention factor for any column pressure drop. The strategy can be implemented either using a mechanistic approach if retention models are known or empirically by iteratively adjusting the co-solvent mass fraction. As a rule of thumb for the empirical approach, we found that the relative mass fraction adjustment needed is proportional to the relative change in the retention factor caused by a change in the pressure drop. Different proportionality constants were required to match retention in the case of increasing or decreasing pressure drops.

Although the use of small sub-2 μm particles in ultra-high-performance liquid chromatography (UHPLC) separations is fully established, the transition from supercritical fluid chromatography (SFC) to ultra-high-performance SFC (UHPSFC), allowing for fast and highly efficient methods, is ongoing.^1^ Berger demonstrated that a 3 × 100 mm column packed with 1.8 μm particles can be used with SFC equipment.^2^ Using the same column with liquid chromatography (LC), the separation would require UHPLC instrumentation. Other authors have demonstrated how existing SFC systems can be reconfigured to allow for very fast and highly efficient chiral separations using sub-2 μm particles.^3^

Considerable effort has been devoted to understanding and explaining the retention shifts due to pressure and temperature effects in method transfer from high-performance LC to UHPLC,^4−6^ especially regarding the quality by design paradigm.^6^ In SFC, these effects are even more complicated as the density of the eluent is much more strongly influenced by pressure than in LC.^7,8^ Therefore, the pressure drop generated in UHPSFC may also cause large variation in retention.

Over the last decade, many researchers have investigated how to quantitatively correlate retention in SFC with state variables such as the pressure, temperature, and composition of the eluent.^7,9−12^ The retention behavior of most solutes eluted in co-solvent-modified SFC has consistently been proven to be the most dependent on the relative amount of co-solvent.^11,12^ One well-known aspect of SFC is the compressibility of fluid, that is, how density varies with pressure and temperature for a certain eluent composition and how this affects retention.^9,13,14^ Most SFC literature studies report the instrument-set volume percentage of co-solvent, a parameter that is notoriously complex to determine and can only be obtained experimentally or, in some cases, numerically.^15,16^ It has repeatedly been shown that the instrument-set volume percentage does not equal the actual value^16,17^ but likely depends on the instrument design and the specific operating conditions. Other studies have characterized retention at a certain mass or mole fraction of co-solvent, which, in contrast to the instrument-set volume percentage, can be readily measured.^10,18−20^ As far as the authors know, no studies reporting co-solvent molarity in SFC studies have been published. From a physical chemistry perspective, adsorption processes are mainly described using solute activities, which are well correlated with molar fractions or molar concentrations.

Method transfer from a 1.7 to a 5 μm particle was shown to decrease the retention factor by over 70% if the method was not adjusted.^21^ Obviously, there is a need to investigate methods to reliably transfer methods from SFC to UHPSFC so that any shifts in retention can be explained and, more importantly, compensated for.^22^ Strategies to match retention in method transfer in analytical and preparative SFC have been proposed.^8,16,18,21^ The most straightforward and efficient approach to matching retention is to adjust the back-pressure regulator of the SFC instrument.^16,21^ However, the approach has limitations: if the average pressure after method transfer requires that the back-pressure regulator be set below its lower limit or above the system pressure limit, the strategy will not work. In addition, it was recently shown that lowering system pressure creates a non-robust separation system,^23^ which clearly demonstrates the need for alternative strategies of method transfer, such as suggested in this study.

The aim of this study is to introduce a new scaling strategy to match any retention shift due to increased or decreased pressure drop during SFC method transfer, for example, from SFC to UHPSFC or from analytical SFC to preparative SFC, without the need to adjust the back pressure. A prerequisite is that we unify the effects of the co-solvent mole fraction and density on retention into one variable, for which we propose the molar concentration (see the “Theory” and “Results and Discussion” sections). This study will be performed by means of simulations using well-characterized experimental sets of data. We will also propose a simplified approach including a practical rule of thumb for empirically applying the new strategy.

## Theory

### Calculating the Methanol Concentration

The molar concentration of methanol in the CO_2_–methanol eluent was calculated according to the relation in eq 1, for which two parameters must be known: (i) the mole fraction of methanol, χ_MeOH_, and (ii) the density, ρ (g L^–1^), of the fluid. Using the experimental setup of Forss et al.,^12^ we measured the mass flow of MeOH and the total flow of MeOH + CO_2_ (the total mass flow were selected because the CO_2_ mass flow were noisier), from which we calculated the density of the fluid using the equation of state of Span and Wagner and the mixing rules of Kunz and Wagner, as implemented in the Reference Fluid Thermodynamic and Transport Properties Database (REFPROP), version 10.^24^ The average column pressure and temperature were used in the calculations.
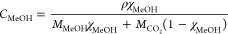
1

A simplified approach that in principle avoids the use of mass flow meters can be performed if the pumps deliver accurate volumetric flow. By knowing the temperature and pressure at each pump head (generally available from instrumentation), the density of the individual fluids can be calculated using REFPROP. From these data, *χ*_MeOH_ can be calculated. Now, at any point in the system where pressure and temperature are known, the density of the mixed fluid and subsequently *C*_MeOH_ can be calculated. However, as this is a fundamental study, no assumptions about the performance of the pumps were considered, and all conditions were measured.

### Empirical Retention Model

In a previous study,^12^ we investigated the dependence of the retention factors of six racemic solutes on the concentration (vol %) of methanol, pressure, and temperature by performing a full-factorial experimental design (see Table S1 in Supporting Information for the particular levels in each design). In the present study, we refitted the experimental data to eq 2 using the methanol molarity instead of the volume percentage. Such a design allows for very accurate prediction of the retention factor within the experimental space.
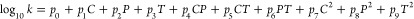
2where log_10_*k* is the logarithm of the retention factor, *p*_0_*–p*_9_ are constants (coefficients), *C* is the molar concentration of methanol (mol L^–1^), *P* is the pressure (bar), and *T* is the temperature (°C). Coefficients were estimated using multiple linear regression, and the regression models were evaluated using analysis of variance. All calculations were performed using MODDE Pro version 11 (Umetrics, Umeå, Sweden).

### Calculation of Apparent Retention Factors and Finding Matching Conditions

**Figure 1:**
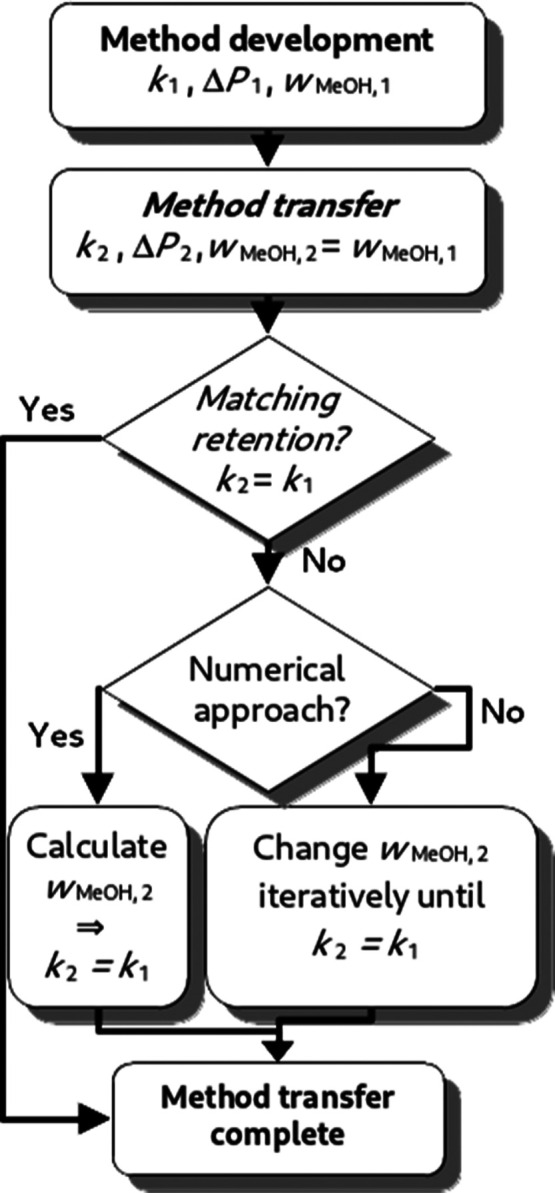
Flowchart describing the steps required to match the retention factor using the molarity-based approach after a method transfer which changes the pressure drop from Δ*P*_1_ to Δ*P*_2_ causing a shift in the retention factor from *k*_1_ to *k*_2_.

For each investigated solute, we used the mass fraction at the center point of the experimental design from the previous study,^12^ also defined in Supporting Information (Figure S1 as the reference condition). Different linear pressure gradients were defined along the column, representing hypothetical pressure drops generated by using different particle sizes at constant mass flow. This scenario is illustrated by the first steps in Figure 1, where a method has been developed and is now transferred to another column, causing a change in the pressure drop. In this case, the assumption of a linear pressure drop is valid at a pressure greater than 130 bar and a temperature below 50 °C.^25^ Here, we also assume that adjusting the mass fraction does not significantly change the viscosity of the mobile phase, which is only true for small variations in the methanol mass fraction. The effect of the mass fraction change on the measured pressure drop was shown to be very small, only a few bar in the investigated mass fraction range, and are summarized in Table S1 in Supporting Information. To avoid extrapolation uncertainties, the pressure gradients were constrained to be within the experimental design, as defined in Table S1 in Supporting Information, and the temperature was set to 32 °C. If experimental retention data below 130 bar is used, the approach that will be presented here would work but the possibility of non-linear pressure drops need to be accounted for. In general, the approach presented here will work regardless of the range of temperature (here the column manufacturer does not recommend operating the column above 40 °C), pressure, and co-solvent as long as the experimental data are available and all gradients in pressure and temperature can be modeled. This is also holds true if the flow rate is changed.

The retention data presented in eq 2 were obtained under near isopycnic and isobaric operation conditions. This information is of limited use since most SFC separations are carried out with pressure drops over the column often exceeding 100 bar. The first step of the calculation involves: (i) interpolating a linear pressure gradient along the column; (ii) calculating the density gradient along the column, ρ(*x*), using REFPROP; and (iii) calculating the methanol molarity gradient along the column. With these data, we can calculate the local retention factor, eq 2, as a function of the position along the column. The values of the parameters in eq 2 are summarized in Table S2 in Supporting Information. By defining the constant total mass flow, , we can also calculate the linear flow rate, *u*(*x*), at the column position *x* as /ρ(*x*). Finally, the apparent retention factor can be obtained from the numerical solution to Supporting Information (eq S4).

The first set of simulations concerns back-pressure-adjusted retention-factor matching and was carried out as follows: two different reference systems were defined, the first with a Δ*P* of 20 bar and the second with a Δ*P* of 80 bar; in both systems, *P*_outlet_ was 160 bar. The mass composition of the fluid was kept constant and identical to the center point of the experimental design for each solute. The temperature was set to 32 °C. The first system was then compared with a system in which the Δ*P* had increased to 80 bar, while the second system was compared with a system in which Δ*P* had decreased to 20 bar. The two systems therefore represent the theoretical effects of either decreasing or increasing the particle size by a factor of 2 while maintaining linear flow and keeping all other conditions constant. Two matching strategies were evaluated: the average-pressure-drop and average-methanol-molarity strategies. The average-pressure-drop system was obtained by changing *P*_outlet_ so that the arithmetic averages of the reference and modified systems matched. To obtain the average methanol concentration, *P*_outlet_ was changed so that the arithmetic mean of the concentration gradient matched that of the reference system.

The second set of simulations concerns the pressure-independent matching strategy in which the methanol mass fraction is adjusted to match the retention. A flowchart describing the steps in this strategy is presented in Figure 1. First, we will describe the numerical approach. Two different reference systems were defined, the first with a Δ*P* of 0 bar and another with a Δ*P* of 100 bar; in both systems, *P*_outlet_ was 140 bar. The retention factor (*k*_1_) of the first system was then compared with the retention factor (*k*_2_) of a system in which Δ*P* had increased to 25, 50, or 100 bar, while the second system was compared with a system in which Δ*P* had decreased to 75, 50, or finally 0 bar. These changes represent the theoretical effects of either decreasing or increasing the particle size versus that of the reference system. The mass composition (*w*_MeOH,1_) of the reference system was identical to the center point of the experimental design for each solute (see Figure S1 in Supporting Information). For each pressure drop, the apparent retention factor was calculated, as described previously, and compared with that of the reference system. Without changing the outlet pressure, the methanol mass fraction (*w*_MeOH,2_) was numerically and iteratively adjusted until the calculated retention factor matched that of the reference system. The selectivity factor was calculated for each system using the mass fraction obtained for matching the retention factor for the first-eluting compound.

From a practical perspective, a pressure-independent matching strategy could be conducted purely experimentally (see Figure 1) by simply iteratively adjusting *w*_MeOH,2_ until retention is matched.

## Results and Discussion

Here, we will introduce the isomolar plot, that is, a plot of the methanol molarity as a function of, for example, pressure or temperature. First, the concept, creation, and implications of the plot will be outlined. We will then explain how to use the plot to understand shifts in the retention factor due to different magnitudes of pressure drop over the column. To do this, we will apply an empirical retention model to six small basic racemic pharmaceutical solutes. Two different scenarios will be investigated: the first concerns an increasing pressure drop due to method transfer from a larger to smaller particle size, and the second concerns a decreasing pressure drop due to method transfer from a smaller to larger particle size (e.g., scale-up in preparative SFC). Finally, a new strategy to match retention to any increase or decrease in pressure drop will be presented, including a rule of thumb for how to adjust the mass fraction depending on the relative retention factor shift.

### Introducing the Isomolar Plot Based on Co-Solvent Molarity

**Figure 2:**
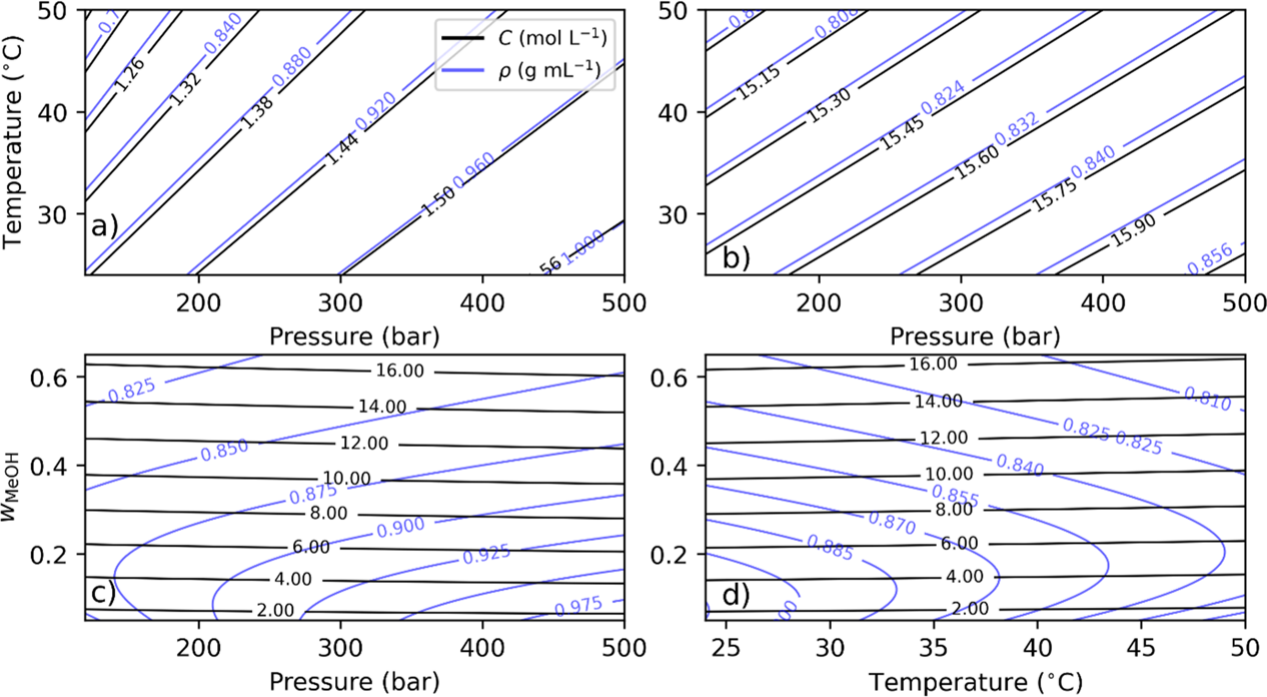
(a,b) Methanol molar concentration (black lines) and density (blue lines) are given for methanol mass fractions of 0.05 and 0.6, respectively. (c) Isomolar plot for varying mass fractions ranging from 0.05 to 0.65 at a fixed temperature of 32 °C. (d) Same as in (c) but at a fixed pressure of 150 bar.

To understand retention shifts in co-solvent-modified SFC for any fluid composition, pressure, and temperature, we propose introducing the isomolar plot. As shown in Figure 2a, the molarity of methanol is plotted versus temperature and pressure for a fixed methanol mass fraction of 0.05. As per definition (eq 1), the concentration is proportional to density, which explains why the contour lines are parallel. The methanol concentration therefore increases with increasing pressure and decreasing temperature. The isomolar plots show that methanol concentration varies nonlinearly with pressure at constant temperature for a 0.05 mass fraction, as indicated by the non-equidistant contour lines (Figure 2a). When increasing the mass fraction to 0.6, the concentration instead varies linearly (Figure 2b). This observation is explained by the compressibility of the fluid, which increases with decreasing mass fraction.

The implication of the isomolar plot is that, from a physical–chemical perspective, the retention cannot be correlated with the mass fraction since the molar concentration changes with pressure. Thus, adsorption and partitioning processes between two phases should, from a fundamental physical–chemical perspective and for ideal systems, best be described using the molarity. For example, the corresponding isomolar plot for water–methanol fluid is presented in Figure S2 in Supporting Information, showing that the co-solvent molarity gradient also exists, but is significantly smaller, in an Reversed Phase Liquid Chromatography (RPLC) system. This implies that describing the retention in RPLC using either the mass fraction or molar concentration is more valid in many cases because both are almost constant over a much larger pressure range. As shown in Figure 2c, the methanol concentration is plotted at a fixed temperature of 32 °C for varying mass fractions and pressures. While Figure 2a,b shows that the variation in the concentration can be determined from the density of the fluid, it is clear that any isopycnic line can be obtained from many combinations of methanol mass fraction and pressure. At certain pressures, identical densities can even be obtained for different mass fractions. As shown in Figure 2d, the methanol concentration is plotted at a fixed pressure of 150 bar for varying mass fractions and temperatures. Here also, any isopycnic line can be obtained from many combinations of methanol mass fraction and temperature; as well, at a certain temperature, identical densities can even be obtained for different mass fractions. This can also be observed in the experimental domain investigated for each solute, where isopycnic lines are not correlated with retention factors (Figure S1 in Supporting Information). From these observations, it follows that retention data in SFC are better correlated with the molar concentration than with density. If an equation of state is unavailable, molarity can be obtained by measuring the mass fraction and density using, for example, inline Coriolis mass flow meters.

### Understanding Pressure-Adjusted Retention Matching for Varying Pressure Drops

To demonstrate the usefulness of the isomolar plot, we will consider two realistic scenarios in SFC concerning method transfer from one column to another in which the column length and mass flow are maintained but the particle size is either reduced by half or doubled. Decreasing particle size would represent a method transfer in order to increase resolution in an analytical separation, for example, going from SFC to UHPSFC. Increasing particle size would represent a method transfer from an analytical to a preparative system, which generally has a lower pressure limit.

In both scenarios, we consider a hypothetical column with an outlet pressure of 160 bar. In the first scenario, the pressure drop over the column increases from a reference of 20 to 80 bar by increasing the inlet pressure from 180 to 240 bar. In the second scenario, the pressure drop decreases from a reference of 80 to 20 bar by decreasing the inlet pressure from 240 to 180 bar. In each scenario, we assume that the mass fraction, mass flow, and temperature remain constant, as supported by the experiments carried out by Forss et al.^12^ and further described in Supporting Information (Table S1). Isomolar plots were generated for the center point for each solute, as defined in Figure S1 in Supporting Information. The apparent retention factor was calculated and the relative change versus the reference pressure drop is presented Figure S3 in Supporting Information. The results show a general decrease in retention by about 10% in the case of increasing pressure drop, whereas an increase in retention by about 10% is observed for the decreasing-pressure-drop cases. Selectivity between the two enantiomers is only marginally affected by either increased or decreased pressure drop. Solutes separated at lower mass fractions display a greater change in retention than do solutes separated at higher mass fractions (for details about exact changes, see Figure S3 in Supporting Information).

**Figure 3:**
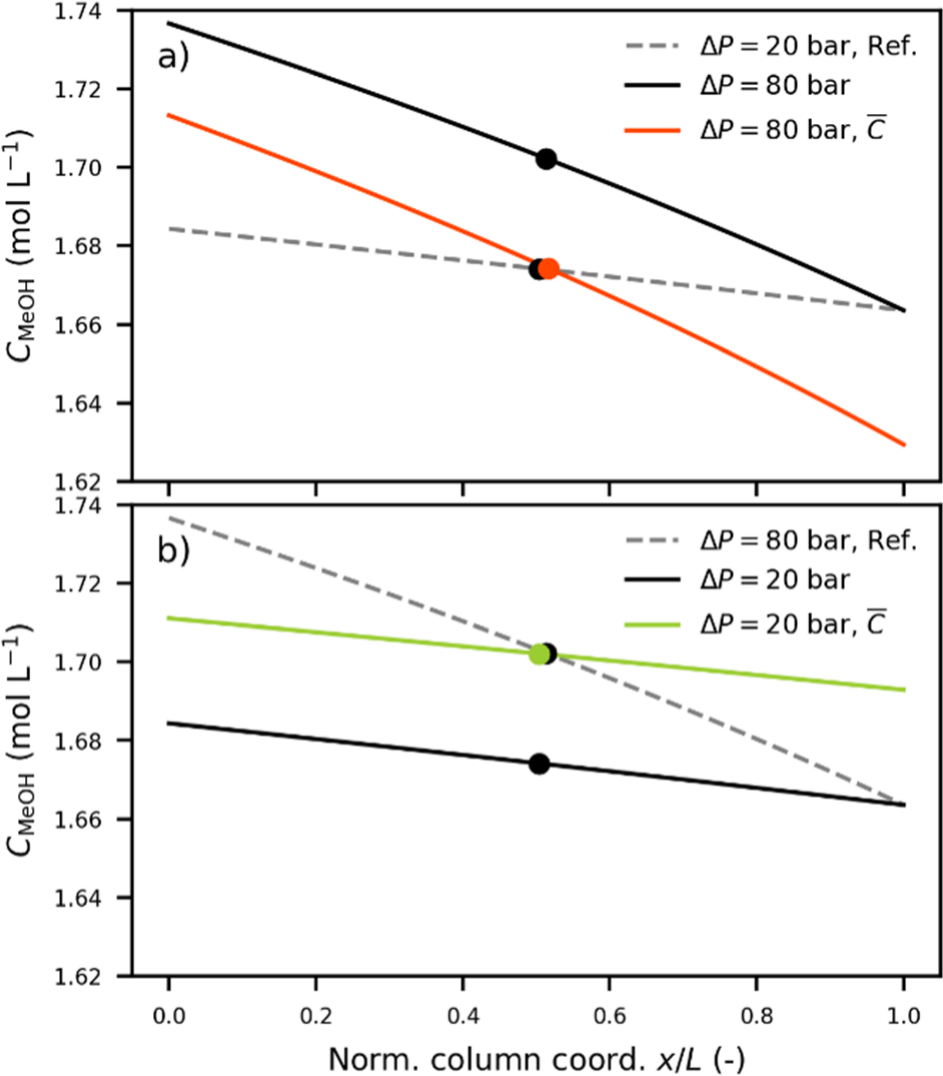
(a,b) Different methanol molar concentration gradients obtained for different pressure drops are illustrated for a mass fraction of 0.061, representing the center point for Clenbuterol. (a) Effects of increasing the pressure drop from 20 to 80 bar at constant versus adjusted outlet pressure, giving the matching arithmetic mean molar concentration (dot) and (b) same comparison for a decreased pressure drop.

To compensate for these observed retention shifts, several authors have introduced the concept of average pressure or average density matching. Average pressure matching relies on decreasing or increasing the system back pressure so that the average pressure drop over the system/column matches that of the reference system.^8,16,21^ Average density matching relies on changing the system back pressure so that the average density matches that of the reference system. Both matching approaches assume a linear pressure drop, which will be assumed here as well. By introducing the isomolar plot, we instead propose the concept of average co-solvent concentration matching. As shown in Figure 3a, the methanol molar concentration gradient along a normalized column coordinate is presented and illustrated under the conditions used for separating the enantiomers of Clenbuterol and Mianserin. The average methanol concentration of the reference system (the black dot on the dashed gray line) is lower than that of the increased-pressure-drop system (the black dot on the solid black line). By lowering the column outlet pressure from the 160 bar of the reference system to 132 bar, the arithmetic average methanol concentration of the 80-bar-pressure-drop system matches that of the 20-bar reference system. It is worth noting that the column outlet pressure adjustment is almost identical to that of average pressure matching, that is, an inlet pressure of 130 bar. Using the same principle for the decreased-pressure-drop system in Figure 3b, we find that increasing the system outlet pressure from 160 to 189 bar will match the average methanol concentration in the case of decreasing the pressure drop from 80 to 20 bar.

**Figure 4:**
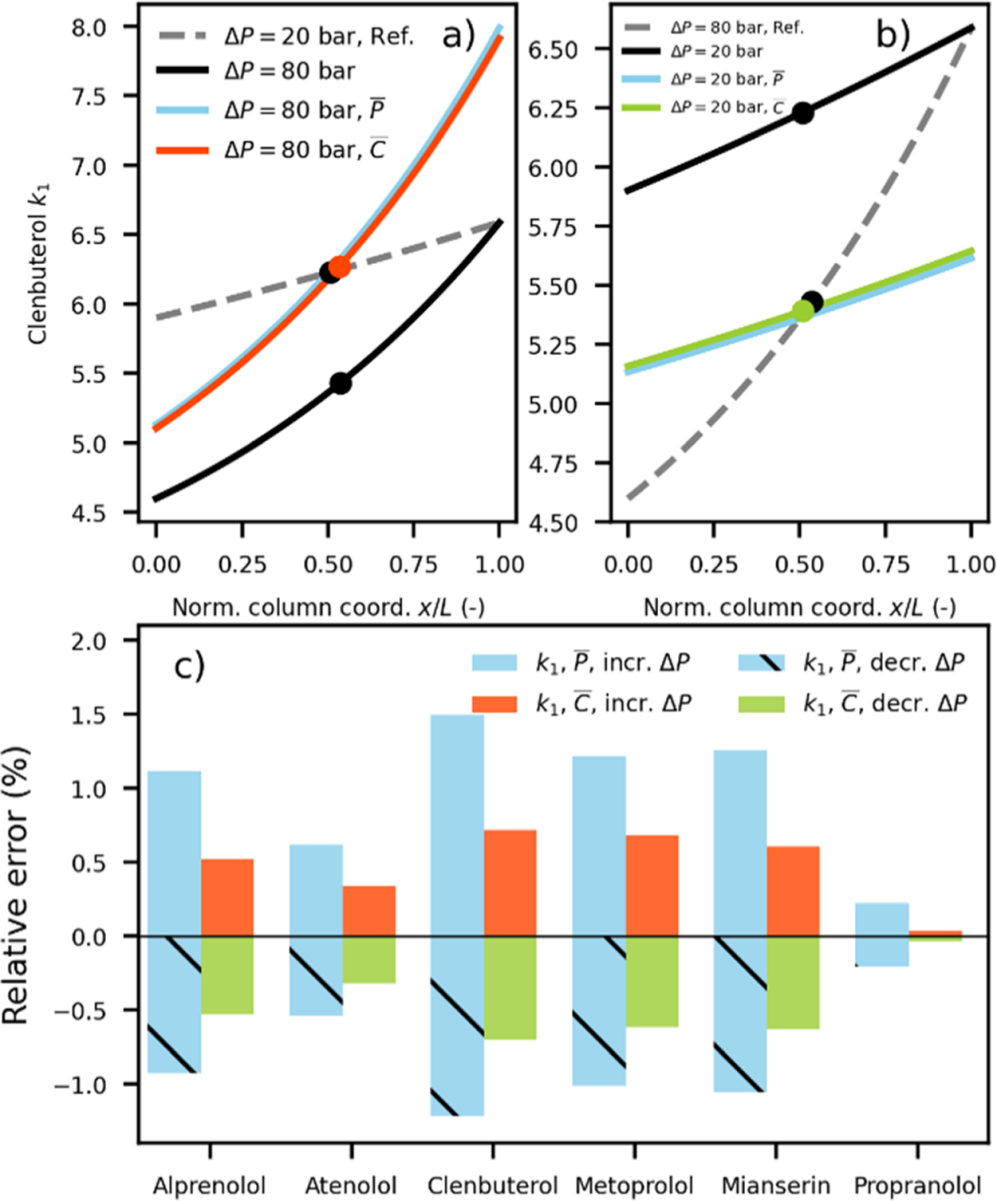
(a,b) Local retention factor, *k*_1_(*x*), of the first-eluting enantiomer of Clenbuterol is plotted together with the apparent (dot) retention factor obtained from simulations using eq S1 for different pressure drops as well as for average pressure or molar concentration matching. (c) Relative error in the apparent retention factor after matching retention using average pressure or concentration.

As described in the “Theory” section, we can calculate the apparent retention factor by solving the mass balance equation (Supporting Information, eq S4 and Page S6) when the local retention factor, *k*(*x*), is known at the normalized column coordinate, *x*. The local retention factor is calculated from eq 2 and is shown for Clenbuterol in Figure 4a,b. Comparing the 20 bar reference pressure drop, as shown in Figure 4a, with the 80 bar pressure drop shows that the calculated apparent retention factor decreases by approximately 15% relative to that of the reference system. Average concentration matching leads to very good agreement with an apparent retention factor only 0.5% greater than that of the reference system. The average-pressure-matching strategy was also very good, with an approximately 1% greater retention factor than that of the reference system. As shown in Figure 4b, the decreased-pressure-drop scenario is shown. Here, the opposite change is observed. The apparent retention factor increases by approximately 15% relative to that of the reference system and average concentration matching worked extremely well, resulting in an apparent retention factor that is only approximately 0.5% smaller than that of the reference system. The average pressure and concentration matching results for all solutes are presented in Figure 4c. The conclusion is that average concentration matching leads to better matching of the retention factor than does average pressure matching. However, the average-pressure-matching strategy is also excellent in this case. In addition, both strategies lead to a slightly higher retention factor in the case of an increasing pressure drop and to a slightly lower retention factor in the case of a decreasing pressure drop.

Clenbuterol displays the largest deviation in the retention factor and Propranolol the smallest. This difference is related to the degree of nonlinearity of the local retention factor gradient and can be understood from two observations. The first is the nonlinearity of the concentration gradient. As can be seen in Figure 3a,b, the nonlinearity increases with increasing pressure drop. The second observation is that the empirical retention model for Clenbuterol predicts that the retention factor will be more strongly dependent on pressure than that in the case of Propranolol (see Figure S4 in Supporting Information). These two observations imply that the error in the average concentration or pressure matching should decrease with the increasing linearity of the concentration gradient. Selectivity changes between the two enantiomers of each solute during average concentration matching are minimal (data not shown). Figure 4. It is important to interpret the present findings in the context of the previous experimental work. Tarafder et al. demonstrated that when performing a method transfer involving a decreased pressure drop (i.e., scale-up) and applying average pressure adjustment, the retention factors of the matched systems were almost always less than those of the reference system.^21^ In another publication, Tarafder et al. demonstrated that average density adjustment led to slightly increased retention in the case of method transfer with an increasing pressure drop.^8^ Both these experimental results are identical to those presented in Figure 4c. Our own study of the effect of retention on a varying flow rate also showed that when the average pressure and average methanol concentration (vol %) were matched, retention in the increased-pressure-drop system was greater than that in the reference system.^16^ The agreement between the present work and independent experimental system results strengthens the validity of the approach presented here.

### Introducing an Alternate Strategy for Retention Matching

**Figure 5:**
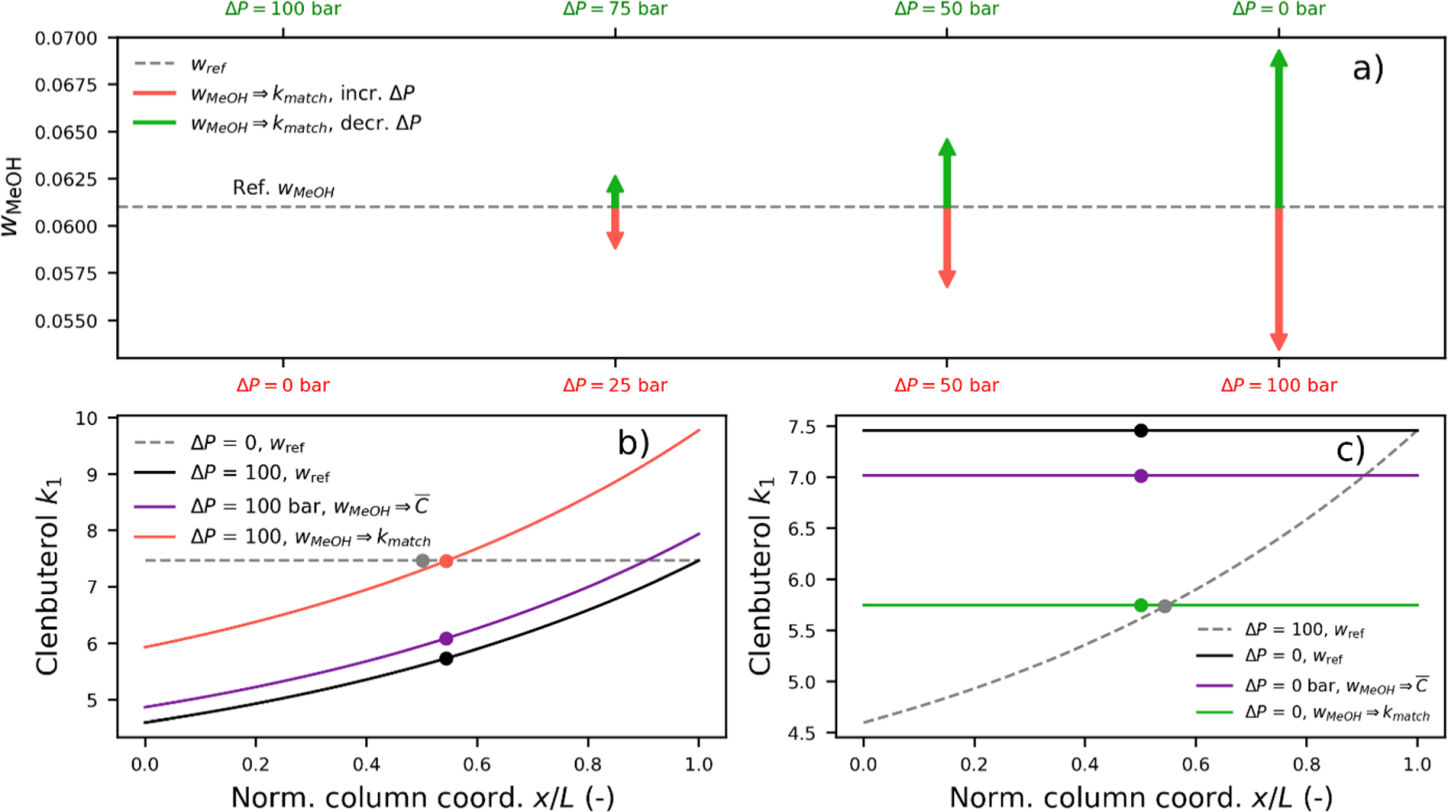
(a) Absolute adjustments of the mass fraction of methanol, *w*_MeOH_, relative to the reference mass fraction of 0.061 required to exactly match the apparent retention factor, *k*_1_, of Clenbuterol when the pressure drop increases (red arrows) or decreases (green arrows) relative to the reference pressure drop. (b,c) Local retention factor plotted with an overlay of the apparent retention factor (dot).

While proven successful and straightforward, pressure-adjusted retention matching in method transfer has limitations. For example, the back pressure can never be adjusted below or above the system’s minimum or maximum pressure limit, respectively, as governed by the back-pressure regulator. More specifically, many systems prevent the user from operating the system with the regulator below approximately 100 bar. Also, current preparative SFC systems typically have upper limits of approximately 300 bar. To overcome the limitations of pressure-adjusted retention matching, we should focus on instead changing the most powerful factor controlling the separation, that is, the co-solvent molarity. From the isomolar plot shown in Figure 2c, it is apparent that to maintain constant methanol molarity given increasing or decreasing pressure, the methanol mass fraction must be either decreased or increased, respectively. However, maintaining isomolar conditions will be insufficient to match retention since each solute also has a pressure dependence that is independent of co-solvent molarity. Therefore, to match retention by adjusting the co-solvent molarity, the adjustment must also compensate for the altered pressure gradient. The magnitude of the mass fraction adjustment needs to be numerically determined by iteratively calculating the apparent retention factor until a mass fraction that gives a matching retention has been found. As shown in Figure 5a, the absolute mass fraction adjustments required to match the reference systems are plotted for Clenbuterol, *k*_1_. The red arrows represent the mass fraction changes for increased pressure drops of 25, 50, and 100 bar relative to that of a 0-bar-pressure-drop reference system. It is apparent that the magnitude of the negative adjustment increases with the increasing pressure drop. The green arrows represent the mass fraction changes for decreased pressure drops of 75, 50, and 0 bar relative to that of a 100-bar-pressure-drop reference system. The origin of these adjustments can be understood from the local retention factor, as plotted in Figure 5b,c. The reference system, as shown in Figure 5a, is represented by the dashed horizontal line, indicating that the apparent retention factor is equal to the local retention factor at any point along the column. Increasing the pressure drop to 100 bar (solid black line) shifts the local retention factor and decreases the apparent retention factor by approximately 20%. By increasing the mass fraction of methanol, *w*_MeOH_, by approximately 15% relative to that of the reference system, we can exactly match the reference system (solid red line). In the other scenario, as shown in Figure 5c, the pressure drop is 100 bar in the reference system and 0 bar in the decreased-pressure-drop system. This difference leads to a 30% increase in the apparent retention factor, which can be exactly matched that of the reference system by decreasing the mass fraction by approximately 15%. As shown in Figure 5b,c, the local retention factor obtained when matching the average molar concentration of the reference system is also presented (see purple lines). Thus, this matching strategy fails to match the apparent retention factor of the reference system due to the independent effect of pressure, as discussed above.

**Figure 6:**
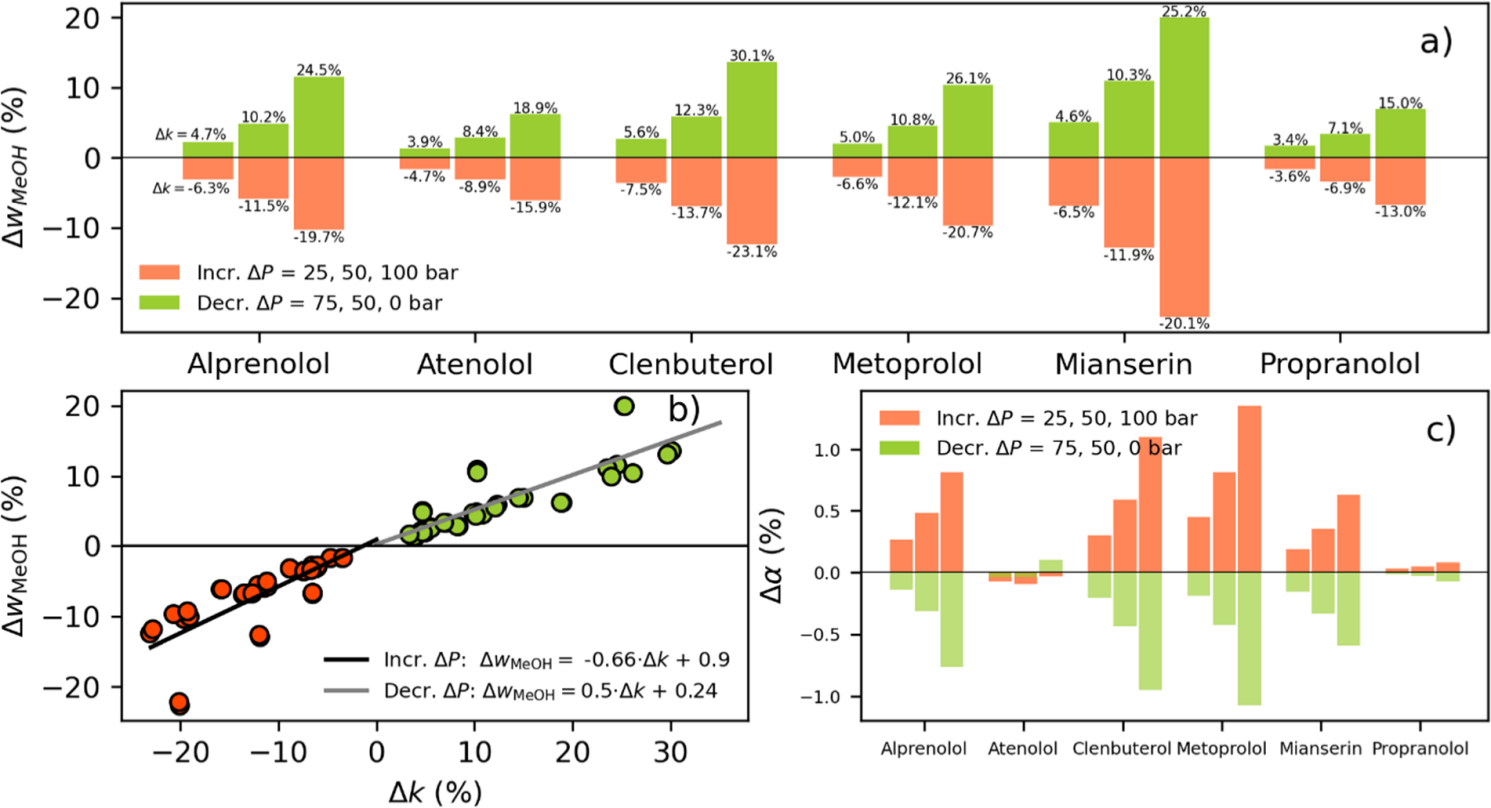
(a) Relative change in the methanol mass fraction required to match a reference system given three different increased- or decreased-pressure-drop scenarios. The relative change in apparent retention before adjustment is noted above the bars. (b) Each adjustment in relative mass fraction to compensate for the relative change in the retention factor (*k*_1_ and *k*_2_) is plotted for both increased and decreased pressure drops. The linear regression for the increased pressure drop is shown by the black line and for the equivalent decreased pressure drop by the gray line. (c) Relative change in the selectivity factor after adjusting the apparent retention factor.

As shown in Figure 6a, the relative mass fraction adjustments required to exactly match the retention during method transfer are presented for all solutes. Increased-pressure-drop systems relative to the 0-bar-pressure-drop system are presented as red bars. Decreased-pressure-drop systems relative to the 100-bar-pressure-drop system are presented as green bars. The relative change in the apparent retention factor resulting from the changed pressure drop is noted above each bar. Comparing the results, we find that the smallest adjustments in the methanol mass fraction are observed for the solutes separated with the highest mass fraction of methanol, that is, Propranolol and Atenolol, which also display the smallest relative changes in the retention factor due to changed pressure drops. On the other hand, we find that solutes separated with the lowest mass fraction of methanol, that is, Clenbuterol and Mianserin, require the largest adjustments and have the largest relative changes in the retention factor. This difference can be understood from the characteristics of the isomolar plot for high and low methanol mass fractions as well as from the fact that solutes have different sensitivities to methanol molarity and pressure (Supporting Information, Figure S4).

A final observation is that an increase in pressure drop always leads to a relatively smaller shift in the retention factor than does an identical decrease in the pressure drop. This is explained by the fact that at higher pressure the fluid is less compressible, resulting in smaller molar co-solvent changes, as can be seen in Figure 2a,b. In conclusion, the mass-fraction-adjustment strategy relies both on the region of the isomolar plot where the experiments are carried out and on the solute-specific dependency on co-solvent concentration, pressure, and temperature.

Finally, a rule of thumb was derived for quantitatively compensating for the retention shift by simply measuring the relative retention shift. We compared the relative retention factor shifts with the corresponding relative mass fraction adjustments for all solutes (*k*_1_ and *k*_2_) and all pressure drops. From these data, we were able to fit a simple linear correlation (see Figure 6b). The correlation shows that a relative decrease in the retention factor when increasing the pressure drop can be compensated for by reducing the methanol mass fraction by approximately 65% of the decrease. On the other hand, a relative increase in the retention factor due to decreased pressure drop can be compensated for by increasing the mass fraction by approximately 50% of the relative increase. Mianserin is a clear outlier, with all its points above or below the linear fit. The general applicability of the rule to different types and sizes of solutes remains to be investigated, but we believe that the rule likely has general validity and can be used as a good first step to compensate for decreased or increased retention.

The selectivity during method transfer is equally important to preserve and was therefore calculated. Changes in the selectivity factor between *k*_2_ and *k*_1_ were calculated from the conditions found to match *k*_1_. As shown in Figure 6c, we observe that no selectivity factor changes by more than approximately ±1.5% for any solute or any pressure drop. Interestingly, the selectivity increases for all increased-pressure-drop systems except Atenolol, while it decreases for the decreased-pressure-drop systems. Nevertheless, the results indicate that mass-adjusted retention matching nearly maintains the selectivity of the separation systems.

## Conclusions

The importance of correlating retention in analytical SFC with parameters based on co-solvent molarity, which depends on both the co-solvent mole fraction and the mobile phase density, was clearly demonstrated. Reporting molarity is an excellent way to standardize SFC studies, regardless of whether they focus on fundamentals, method development, method transfer, or preparative separations.

Based on this conclusion, we introduced the so-called isomolar plot, which describes the molar concentration as a function of the pressure, temperature, and mole fraction of methanol. The isomolar plot was first used to understand retention shifts for six racemic basic solutes due to increasing or decreasing pressure drops caused by changing the particle size and/or flow rate during method transfer. The approach of adjusting the back pressure to match the arithmetic-mean pressure drop is successful because of its combined effect of matching both the pressure and molar concentration. By adjusting the pressure to match the average molar concentration, we could demonstrate slightly better retention matching. Neither approach can exactly match retention.

Finally, we proposed and elucidated a new strategy to compensate for any retention shift due to varying pressure drops during method transfer from SFC to UHPSFC, without the need to adjust pressure. The strategy instead involves adjusting the co-solvent mass fraction of an increased- or decreased-pressure-drop system until the apparent retention factor exactly matches that of the reference system. This approach was shown to exactly match retention and only marginally affect the selectivity factor and does not require any adjustment of the back-pressure regulator, which cannot always be done when going from SFC to UHPSFC.

As a rule of thumb, if the pressure drop relative to that of the reference system increases, the mass fraction should decrease by a proportionality constant of approximately 0.7 relative to the decrease in the retention factor. If the pressure drop instead decreases, the mass fraction should increase by a proportionality constant of approximately 0.5. These proportionality constants were proven valid for a set of six racemic solutes separating 5–30 vol % methanol on a chiral stationary phase.

The work presented here will be useful for two reasons: first, it correlates the retention mechanisms in SFC with molarity, which is a fundamental property in all equilibrium theory from a physical chemistry perspective; second, it gives practitioners a universal tool for performing method transfer in SFC.

## Supporting Information Available

Experimental domain for each solute; corresponding experimental domain response surfaces as a function of the mass fraction of MeOH and pressure; design of experiment raw model term coefficients including a scaled and centered coefficient plot; numerical solution of the column mass balance model used to calculate retention factors; isomolar plot of methanol–water; and relative change of retention factors and selectivity during method transfer (PDF)
