# Supplementary material for: A Retention-Matching Strategy for Method Transfer in Supercritical Fluid Chromatography: Introducing the Isomolar Plot Approach
Source: Anal Chem. 2021 Apr 12;93(16):6385–93. doi: 10.1021/acs.analchem.0c05142 (PMC8153393; doi:10.1021/acs.analchem.0c05142)
Supplement: Supplementary file 1 — ac0c05142_si_001.pdf [file ac0c05142_si_001.pdf]

Supporting Information:

A Retention-Matching Strategy for Method Transfer in SFC: Introducing the Isomolar Plot Approach

Martin Enmark, Jörgen Samuelsson* and Torgny Fornstedt*

Department of Engineering and Chemical Sciences, Karlstad University, SE-651 88 Karlstad, Sweden

** Corresponding authors:*
Phone: +46 54 700 1960; E-mail: Torgny.Fornstedt@kau.se; https://orcid.org/0000-0002-7123-2066
Phone: +46 54700 1920; E-mail: Jorgen.Samuelsson@kau.se; https://orcid.org/0000-0003-1819-1709

Contents

Table S1 2
Figure S1 4
Table S2 5
Numerical Solution of the Column Mass Balance Model, Equation S4 6
Figure S2 7
Figure S3 8
Figure S4 9
References 10

Design of Experiments Raw Data

Table S1. List of the experimental design parameters, i.e., set co-solvent v%, pressure, and temperature, for each investigated solute from unpublished data from Forss et al.[1] The cells show the measured min/max mass fractions at all pressures and temperatures investigated per solute, as well as the average measured pressure and temperature at each set v%**.**

			Set pressure (bar)			Set temperature (°C)		
			120	180	240	24	32	40
		w_{MeOH} (min/max)	Avg. measured			Avg. measured		
Alprenolol								
Set v%	5	0.042/0.044	127.312	188.829	250.031	23.695	31.221	38.712
	7.5	0.062/0.067	127.447	188.971	250.078	23.736	31.218	38.735
	10	0.084/0.088	127.587	189.018	250.178	23.756	31.207	38.714
Atenolol								
Set v%	15	0.127/0.133	128.407	190.004	251.398	23.769	31.284	38.772
	20	0.173/0.181	128.949	190.568	251.931	23.782	31.303	38.818
	25	0.218/0.228	129.602	191.158	252.627	23.853	31.355	38.805
Clenbuterol /Mianserin								
Set v%	5	0.043/0.045	127.545	189.188	250.459	23.79	31.433	39.085
	7	0.059/0.063	127.591	189.244	250.488	23.793	31.445	39.091
	9	0.077/0.08	127.791	189.312	250.623	23.803	31.438	39.088
Metoprolol								
Set v%	5	0.044/0.046	127.421	188.82	249.85	24.029	31.493	39.154
	10	0.088/0.092	127.848	189.121	250.253	24.453	31.484	39.217

	15	0.132/0.138	128.184	189.613	250.727	24.305	31.468	39.135
Propranolol								
Set v%	15	0.129/0.136	128.066	189.641	250.878	24.029	31.493	39.154
	22.5	0.199/0.205	128.98	190.504	251.743	24.453	31.484	39.217
	30	0.269/0.276	130.105	191.62	252.855	24.305	31.468	39.135

Design of Experiments – Experimental Domain Response

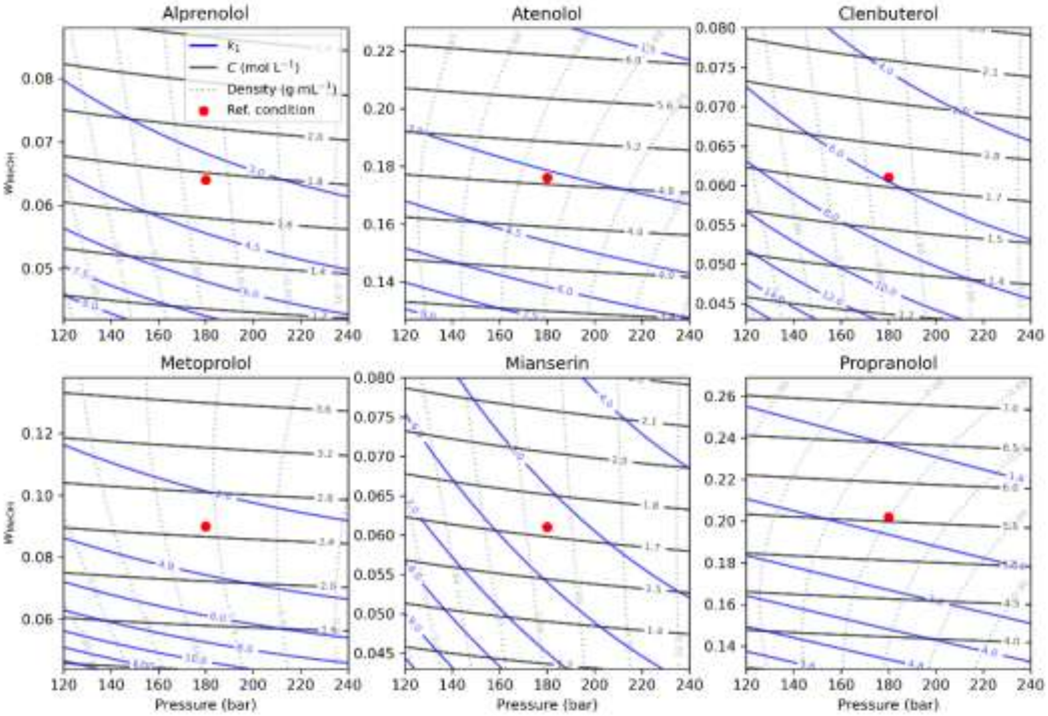

Figure S1. Plot of the apparent retention factor, k_1 (black lines), for each solute throughout the design space of the methanol mass fraction and pressure at the center-point temperature of 32°C. The red dots indicates the center-point/reference mass fraction used throughout the study. The blue lines represent the molarity of MeOH in the mobile phase.

Design of Experiments – Raw Coefficients

Table S2. Significant raw model term coefficients from the fitted DoE model eq. 2 used for all calculations of local and apparent retention factors; NS = non-significant parameter.

	Const.	*P*	C_m	*T*	P^2	C_m^2	T^2	$P \cdot C_m$	$P \cdot T$	$C_m \cdot T$
**Alprenolol** k_1	2.12E+00	-2.02E-03	-1.02E+00	4.47E-03	4.82E-06	1.53E-01	NS	NS	-3.38E-05	NS
k_2	2.35E+00	-1.84E-03	-1.04E+00	1.17E-03	4.43E-06	1.57E-01	NS	NS	-3.30E-05	NS
**Atenolol** k_1	2.92E+00	-2.42E-03	-4.89E-01	-1.34E-02	2.28E-06	2.26E-02	1.26E-04	9.61E-05	NS	NS
k_2	3.10E+00	-2.09E-03	-4.63E-01	-1.09E-02	2.65E-06	2.27E-02	9.08E-05	7.54E-05	-1.12E-05	-6.22E-04
**Clenbuterol** k_1	2.37E+00	-2.88E-03	-1.08E+00	8.51E-03	5.95E-06	1.58E-01	NS	2.05E-04	-4.19E-05	NS
k_2	2.68E+00	-3.77E-03	-1.09E+00	NS	-5.84E-06	1.70E-01	NS	NS	NS	NS
**Metoprolol** k_1	2.32E+00	-3.60E-03	-7.67E-01	NS	5.14E-06	7.19E-02	NS	9.58E-05	NS	NS
k_2	2.84E+00	-2.43E-03	-8.12E-01	-3.91E-03	4.28E-06	8.27E-02	NS	9.98E-05	-2.30E-05	NS
**Mianserin** k_1	2.14E+00	-2.71E-03	-4.48E-01	-1.82E-02	6.30E-06	5.99E-02	3.28E-04	3.23E-04	-5.52E-05	-1.30E-03
k_2	2.22E+00	-2.77E-03	-4.44E-01	-1.93E-02	6.31E-06	5.84E-02	3.40E-04	3.18E-04	-5.29E-05	-1.50E-03
**Propranolol** k_1	1.88E+00	-9.55E-04	-2.72E-01	-3.35E-03	NS	1.12E-02	8.92E-05	9.67E-05	-1.80E-05	-9.92E-04
k_2	2.20E+00	-1.06E-03	-2.90E-01	-3.46E-03	NS	1.24E-02	NS	1.10E-04	-1.59E-05	-7.62E-04

Numerical Solution of the Column Mass Balance Model, Equation S4

We model the separation process using a one-dimensional lumped kinetic model (LKM). [2] Each compound transported in the column, denoted below by index *i*, is described by an ordinary differential equation:

$$\frac{\partial c_i(x,t)}{\partial t} + u\frac{\partial c_i(x,t)}{\partial x} = D_i\frac{\partial^2 c_i(x,t)}{\partial x^2} - \frac{k_i}{\epsilon}({q_i}^* - q_i)$$
$$\frac{\partial q_i(x,t)}{\partial t} = \frac{k_i}{1-\epsilon}({q_i}^* - q_i) \tag{S1}$$

where ϵ is the fraction of total volume occupied by the mobile phase, c_i the concentration of compound in the mobile phase, u the linear velocity of the mobile phase, and D_i the lumped diffusion coefficient; q_i is the concentration of adsorbed compound in the stationary phase and ${q_i}^*$ the equilibrium concentration in the stationary phase determined by the adsorption isotherm. In this case, a linear adsorption isotherm was used and was defined as $q = k(P, T) / F$, where k is taken from eq. (2) and F is the phase ratio defined as $(1 - \epsilon)/\epsilon$; k_i is a forcing constant that determines the timescale in which the equilibrium between the mobile and stationary phases is reached. The above model is solved using the Danckwerts boundary conditions at the inlet, $uc_i - D_i\frac{\partial c_i}{\partial x} = uc_i^{inj}(t)$, and neutral boundary conditions at the outlet, $\frac{\partial c_i}{\partial x} = 0$, with $c_i^{inj}(t)$ indicating the injected concentration of compound *i*. The LKM model is an extension of the equilibrium dispersive (ED) model of chromatography, allowing the adsorbed concentration, q_i, to be in equilibrium with the isotherm ${q_i}^*$. The ED model is recovered by eliminating the term $({q_i}^* - q_i)$ in the above system of two equations.

We solve the system of equations in the above form by discretizing it using an up-winding finite-volume method, which reduces the system of partial differential equations to a system of ordinary differential equations. The resulting system is solved with an implicit integrator, because it typically behaves like a stiff problem, using Radau IIA methods implemented in Scipy.

Isomolar Plot Methanol–Water

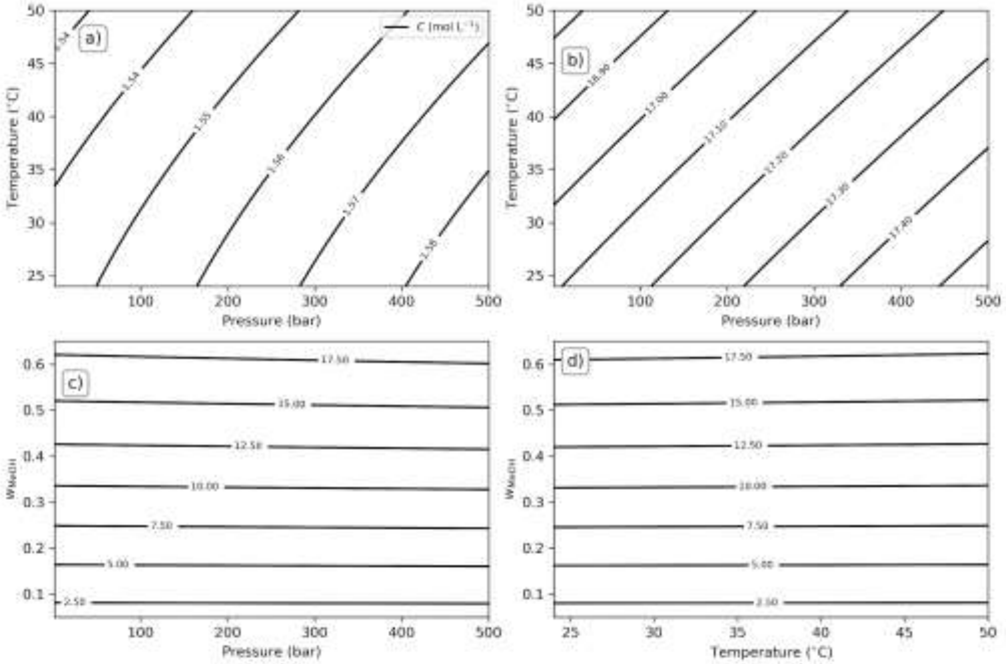

Figure S2. (a, b) Methanol molarity dependence on temperature and pressure for methanol mass fractions of 0.05 and 0.6 methanol in water. (c) Isomolar plot for varying mass fractions ranging from 0.05 to 0.65 at a fixed temperature of 32°C. (d) The same as in (c) but at a fixed pressure of 150 bar.

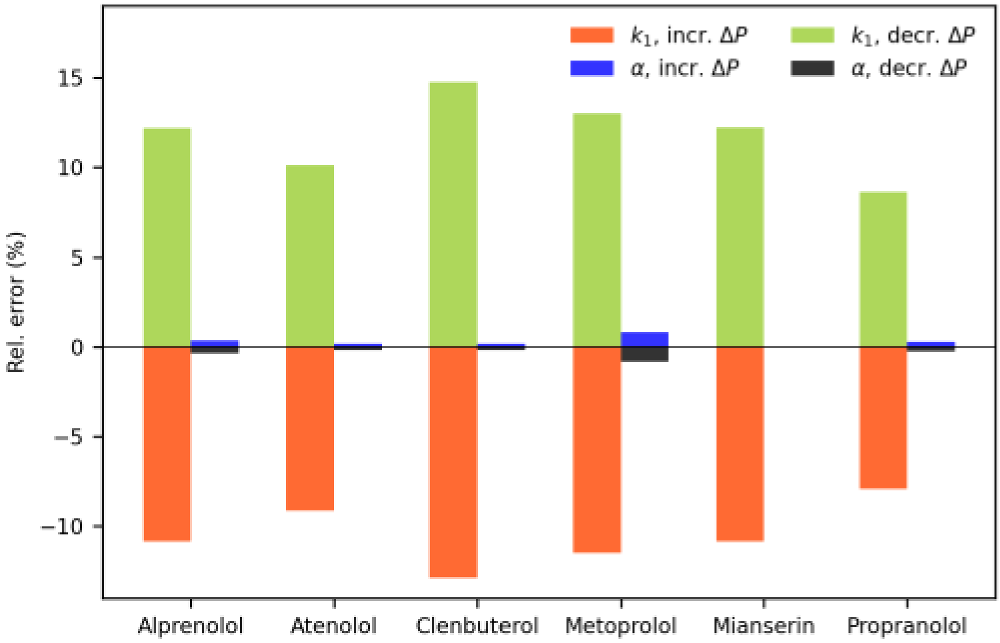

Figure S3. The relative effect on the retention and selectivity factors on all solutes for the increased- and decreased-pressure-drop systems described in section "**Understanding Pressure-adjusted Retention Matching for Varying Pressure Drops**"

Design of Experiments – Scaled and Centered Coefficients

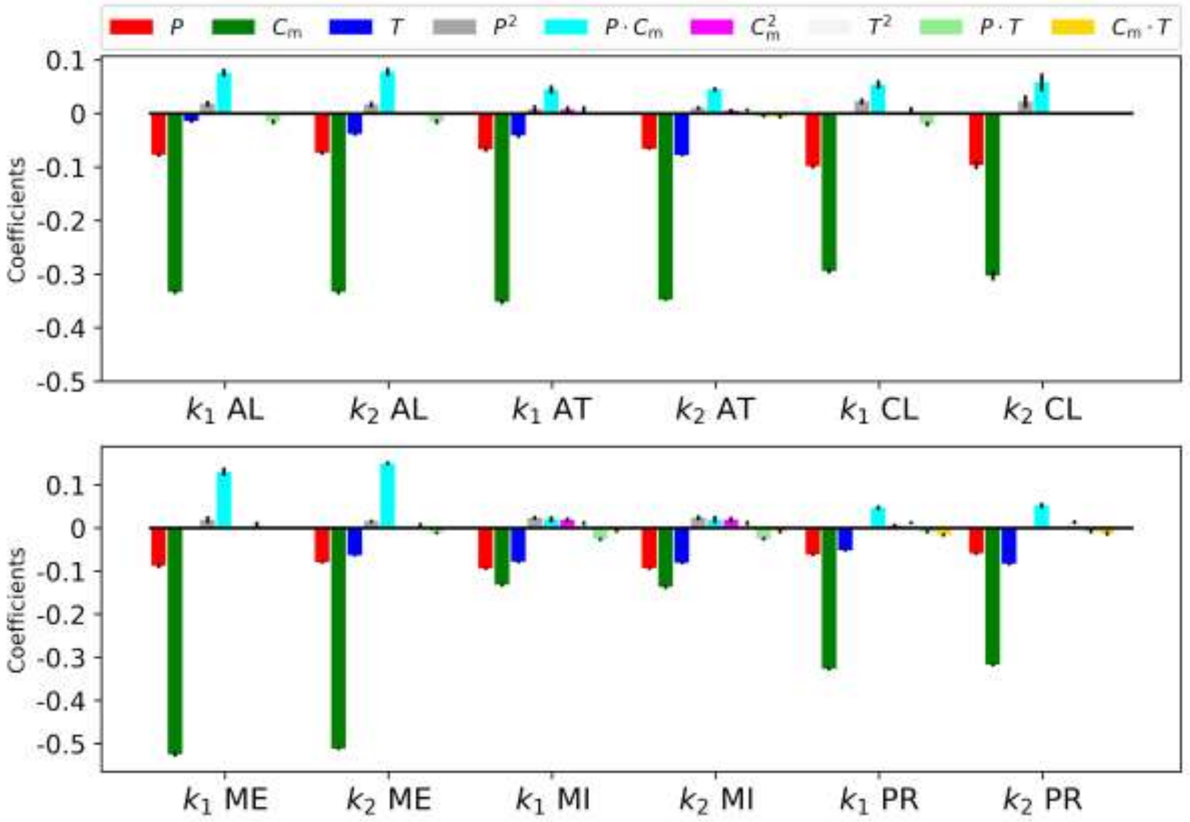

Figure S4. Scaled and centered coefficients of k_1 and k_2 for all solutes: Alprenolol (AL), Atenolol (AT), Clenbuterol (CL), Metoprolol (ME), Mianserin (MI), and Propranolol (PR).

References

(1) Forss, E.; Haupt, D.; Stålberg, O.; Enmark, M.; Samuelsson, J.; Fornstedt, T. Chemometric Evaluation of the Combined Effect of Temperature, Pressure, and Co-Solvent Fractions on the Chiral Separation of Basic Pharmaceuticals Using Actual vs Set Operational Conditions. *J. Chromatogr. A* **2017**, *1499*, 165–173. https://doi.org/10.1016/j.chroma.2017.03.077.

(2) Guiochon, G.; Shirazi, D. G.; Felinger, A.; Katti, A. M. *Fundamentals of Preparative and Nonlinear Chromatography*, 2nd ed.; Academic Press: Boston, MA, 2006.
